# Short term clinical outcomes of Everolimus-eluting stents in patients with stable angina pectoris

**DOI:** 10.12669/pjms.342.13770

**Published:** 2018

**Authors:** Muhammad Habeel Dar, Yasir Adnan, Mohammad Faheem, Imran Khan, Lubna Noor

**Affiliations:** 1 Dr. Muhammad Habeel Dar, FCPS Cardiology. Department of Cardiology, Maulvi Ameer Shah Hospital, Peshawar, Pakistan; 2 Dr. Yasir Adnan, FCPS. Department of Cardiology, Police and services Hospital, Peshawar, Pakistan; 3 Mohammad Faheem, FCPS Cardiology. Department of Cardiology, Khyber Teaching Hospital, Peshawar, Pakistan; 4 Imran Khan, FCPS Cardiology. Department of Cardiology, Hayat Abad Medical Complex, Peshawar, Pakistan; 5 Dr. Lubna Noor, FCPS Cardiology. Department of Cardiology, Lady reading Hospital, Peshawar, Pakistan

**Keywords:** Everolimus eluting stents, stable angina, myocardial infarction, target vessel revascularization

## Abstract

**Background & Objective:**

Everolimus-eluting stents, compared with bare metal stents, reduced the risk of restenosis in clinical trials with strict inclusion and exclusion criteria. The objective of this study was to determine the three months clinical outcomes of Everolimus Eluting Stents in patients with stable angina pectoris in Pakistani population.

**Methods:**

It was a descriptive cross-sectional study and the data was collected from Catheterization Laboratory Cardiology Department Lady Readings Hospital Peshawar. Our study included all the patients with stable coronary artery disease who had received Everolimus eluting stents from August, 2013, to April, 2014. Total study duration was 09 months. The primary end points were the rate of target vessel revascularization, myocardial infarction at three months. All those patients who received Everolimus coronary stents were recalled after three months from the index procedure and enquired about target vessel revascularization (TVR), myocardial infarction and hospitalization over the last three months. Data analysis was done using SPSS version 16.

**Results:**

Our study included 378 patients with stable ischemic heart disease who underwent revascularization with Everolimus eluting stent. These patients were followed up for a period of 3 months for target vessel revascularization(TVR) and myocardial infarction(MI). Mean age was 57.04±9.307, males were (72%). Left Anterior Descending (LAD) and Left circumflex (LCx) were the predominant vessels vascularized. Mean length of Everolimus eluting stent was 21.91± 4.6 while mean diameter of stent was 2.90±0.248. Thirteen (3.4%) patients had TVR and 14 (3.7%) patients had MI during three months follow up after PCI. TVR and MI were prevalent in patients who received longer Everolimus stents as compared to those who received shorter stents at three months, and the difference between the two was statistically significant.

**Conclusion:**

Short-term results from this study suggest that real-world outcomes among 378 patients are comparable to those reported in other registries and trials, and safety outcomes as measured by rates of TVR, MI were low. The long-term safety of Everolimus-eluting stents needs to be ascertained in large, randomized trials.

## INTRODUCTION

Coronary heart disease is an international health problem in both men and women and is the leading cause of death in the developed countries.^1^ The prevalence is equally high in South Asia including Pakistan.^1^ This is about 11.2% in our local population and is more prevalent in females (13.3%) than males (7.9%).^2^

There is lower incidence of target vessel revascularization in patients treated with Drug Eluting stents (DES) as compared to Bare metal stents (BMS).^3^ There are concerns regarding the safety of first generation DES, as they may lead to delayed healing and endothelial dysfunction of the stented arterial segment, increasing the risk of late thrombotic events.^4^ Second-generation DESs have better safety and efficacy. Recent randomized trials with head-to-head comparisons of the first-generation paclitaxel-eluting TAXUS stent (PES) (Boston Scientific, Natick, MA, USA) with second-generation stents suggest that the newer DESs are more effective and are safer^5^-^7^ Various studies have shown that DES in comparison to BMS reduces the risk of restenosis and major adverse cardiac events including target vessel revascularization.^8^

In terms of permanent polymer DES, the Everolimus Eluting Stent (EES) represents a potential step forward in stent technology. In a number of randomized controlled studies, it has proven superior to the first-generation paclitaxel-eluting stent.^6^-^7^ Studies show 12 months clinical outcomes for Everolimus Eluting Stents (EES) as target vessel revascularization (4.8%) and myocardial infarction (4.1%).^9^ Our objective was to determine the three months clinical outcomes of Everolimus Eluting Stents in patients with stable angina pectoris in Pakistani population

## METHODS

It was a descriptive cross-sectional study and the data was collected from Catheterization Laboratory Cardiology Department Lady Readings Hospital Peshawar. Our study included all the patients with stable coronary artery disease who had received Everolimus eluting stents from August, 2013, to April, 2014. Total study duration was 09 months. The primary end points were the rate of target vessel revascularization, myocardial infarction at three months Patients of stable angina pectoris was diagnosed based on history of chest, jaw or arms pain on exertion and positive exercise tolerance test for ischemia or angina using Bruce or modified Bruce protocol using Quinton Q-Stress TM 55 machine.

### Target Vessel Revascularization (TVR)

TVR was defined as any repeat percutaneous intervention or surgical bypass of any segment of the target vessel.

### Myocardial Infarction (M.I)

Myocardial infarction was defined as presence of typical chest pain with either ST elevation of more than 1mm in two consecutive leads on ECG or positive cardiac biomarkers. If M.I happened to the patient who has been stented, it is easily diagnosed on E.C.G leads which show specific leads for specific coronary arteries.

Patients underwent percutaneous coronary intervention in the Lady Reading Hospital Catheterization Suite by experienced interventionists with minimum of 5 years of experience, using catheterization machine Axiom Artis (flate panel) Siemens.

All patients who received Everolimus coronary stents for stable angina pectoris were followed up three months after the index procedure for the primary outcomes. All the data collected with the help of proforma were entered and analyzed SPSS version 16.

## RESULTS

All 378 patients were enrolled in this study from August 2013 to April 2014. Patients were followed up for three months. Mean age was 57.04 ± 9.307. Male were 272 (72%) while female was 106 (28%). Majority of the patients had revascularization of LAD and LCx with DES. Mean length of drug eluting stent was 21.9±4.6 while mean diameter of stent was 2.90±0.2483. Table-I

**Table-I:** Patient’s demographic data and angiographic Characteristics. (N = 378).

Age	57.04±9.307
Male	72% (272)
*Target Vessel*	
LAD	47.1% (178)
LCX	27.8% (105)
RCA	6.3% (24)
LAD and LCX	9.8% (37)
LCX and RCA	6.1% (23)
LAD and RCA	2.9% (11)
Mean length of Stent	21.899±4.600
Mean diameter of Stent	2.902±0.248

**Table-II:** Clinical outcomes of patients.

Variables	Myocardial infarction	Target vessel Revascularization.
*Gender*		
Male	9(3.3%)	9(3.3%)
Female	5(4.7%)	4(3.8%)
P-Value	0.548	0.762
*Age of the patients*		
<60 60 years	4(2.7%)	4(2.7%)
60-85 years	10(4.3%)	9(3.9%)
P-value	0.579	0.773
*Stent Vessels*		
LAD	8(4.5%)	5(2.8%
LX	0	1(1%)
RCA	1(4.2%)	0(0%)
LAD & CX	2(5.4%)	7(18.9%)
CX & RC	2(8.7%)	0(%)
LAD & RC	1(9.1%)	0(%)
P-value	0.208	0.000

Primary end points included Target vessel revascularization and Myocardial infarction at three months. Out of 378 patients 14 (3.7%) patients suffered from MI during three months follow up. About 13 (3.4%) patients had Target vessel revascularization during follow up.

The three months’ clinical outcomes were stratified among male and female patients. At three months, rate of myocardial infarction showed no statistical difference between male 3.3% and female 4.7% with p *= 0.548*. The 3-months event rate of target vessel revascularization (TVR) was slightly lower in the male group 3.3% versus female group 3.8% with *p=0.762* which is statistically not significant. Patients were divided into two categories, age <60 years and age 60-85 years. The rate of myocardial infarction was slightly higher in older age group (4.3%) as compared to younger age group (2.7%), statistically non-significant. Similarly, more patients suffered target vessel revascularization in older age group 3.9% as compared to patients in younger age group 2.7%, also statistically non-significant. The Right coronary artery and left circumflex artery when taken together, had the highest propensity to develop myocardial infarction (8.7%), whereas rate of target vessel revascularization (TVR) was slightly higher in patients who had Left anterior descending artery and Left circumflex artery stented (18.9%).

**Fig.1:**
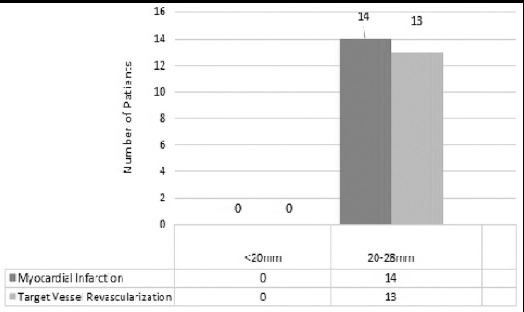
Effect of stent length on clinical outcomes.

As the stented length increased, there was a trend toward higher (statistically significant) rates of clinical outcomes. Fig.1.

**Fig.2:**
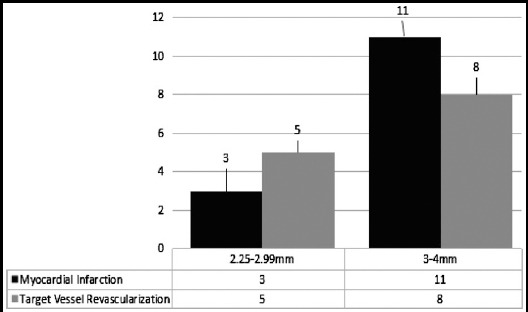
Effect of stent diameter on clinical outcomes.

The event rate of selected outcomes for stented diameter at three months is shown for EES in Fig.2. For EES there were no clear relationships between stented diameter and outcome for any of the clinical outcome variables.

## DISCUSSION

The EES is based on cobalt-chromium multilink vision stent platform. The SPIRIT (I-IV) series of trials have demonstrated the efficacy of the ESS in comparison with the PES. The largest scale SPIRIT (IV) trial was powered to assess superiority of ESS over PES. The 2-year follow-up revealed significantly reduced rates of TVR and MI for EES.^7^ Our report describes 3-months data of clinical outcomes of the Everolimus Eluting Stents. These results, obtained from 378 patients, provide compelling evidence for the safe and effective use of the EES in routine clinical practice. The results are similar to those reported in randomized clinical trials in the past.^5^-^8^ all reported events were adjudicated.

In our study rate of MI was 3.7% and rate of TVR was 3.4%. The rates were slightly lower in our study as compared to previous randomized trials^8^. However, it is worth noting that in our study we excluded patients with TVD and those with complex lesions such as Left main stem disease and previous history of revascularization whether percutaneous coronary intervention or coronary artery bypass graft which might be the reason for slightly better outcomes.

In our study 14(3.7%) patients developed myocardial infarction which was lower than observed in previous study. In one large, randomized trial the rate of MI was 4.1% at one year.^8^ As we followed the patients for initial three months when the incidence of MI is low and beyond three months after deployment of EES, the incidence of MI increases. Data from post hoc analyses,^10^ meta-analyses^11^-^12^ and registries^13^ have suggested an increased risk of adverse ischemic events at long-term follow-up possibly related to stent thrombosis in patients treated with Drug-eluting stent such as Everolimus-eluting stent (hence the recent aphorism ‘trading restenosis for thrombosis’). Another reason for lower rate of MI in our study, might be the exclusion criteria. We excluded all the patients with triple vessel disease and those with previous history of revascularization in the form of PCI or CABG. So, by excluding patients with complex lesion, lower rates of clinical outcomes were predicted.

The event rate for Target vessel revascularization 13(3.4%) in our study which was slightly lower than the previous studies. In one of the previous trial comparing Zotarolimus-eluting stents with Everolimus-eluting stents, the incidence of Target vessel revascularization was 4.8% at one year in EES.^8^ As mentioned earlier our follow up was for just three months and we also excluded patients with complex lesion, which might be the reason for better outcome in this study. The frequency of target vessel revascularization was highest 18.9% for those patients having double vessel disease and stented to LAD and CX at the same time. Severity of disease in these arteries and occurrence of disease in two arteries at the same time, might be the explanation of higher rates of events in these patients.

We also found that rate of clinical outcomes was slightly lower in male as compared to female patients, but it was statistically not significant. Clinical outcome of women after PCI has been challenging. Studies have shown that female sex has been associated with increased complications during and after PCI due to more comorbid conditions.^14^-^15^

It was observed that older patients had relatively higher rates of MI, TVR at three months follow up, though it was statistically not significant. Elderly patients usually have multivessel disease. They also tend to have diffused atherosclerosis in their coronaries along with other comorbid conditions.

We evaluated the rate of selected outcomes at three months for length of Everolimus-eluting stents (EES). There were higher rates of MI and TVR in the longest stented length compared with the shortest stented length at three months, which were statistically significant. We also noticed that for EES there were no clear relationships between stented diameter and outcome for any of the clinical outcome variables. Stent length and diameter have been demonstrated to have an important effect on restenosis and rates of target-vessel revascularization (TVR) with bare-metal stents (BMS).^16^,^17^ Drug-eluting stents (DES) such as (EES) have been shown to reduce restenosis and TVR compared with BMS across a wide spectrum of stent lengths and diameters. One recent trial showed that for EES, there were no significant differences in any of the clinical outcomes among the different stented length and diameter.^18^

## CONCLUSION

Short-term results from this study suggest that real-world outcomes among 378 patients are comparable to those of the previous studies. These 3-month results clearly provide evidence for the safety and effectiveness of the EES for patients with stable angina and are consistent with those reported in the previous trials. For long-term safety of Everolimus-eluting stents, further research with better study design is required.

### Authors’ Contribution

**MHD** conceived, designed and did statistical analysis.

**MF** did editing of manuscript.

YA and IK did data collection and manuscript writing

**LN** did review and final approval of manuscript.
